# Anti-Transcription Factor RNA Aptamers as Potential Therapeutics

**DOI:** 10.1089/nat.2015.0566

**Published:** 2016-02-01

**Authors:** Estefanía Mondragón, Louis James Maher

**Affiliations:** Department of Biochemistry and Molecular Biology, Mayo Clinic College of Medicine, Rochester, Minnesota.

## Abstract

Transcription factors (TFs) are DNA-binding proteins that play critical roles in regulating gene expression. These proteins control all major cellular processes, including growth, development, and homeostasis. Because of their pivotal role, cells depend on proper TF function. It is, therefore, not surprising that TF deregulation is linked to disease. The therapeutic drug targeting of TFs has been proposed as a frontier in medicine. RNA aptamers make interesting candidates for TF modulation because of their unique characteristics. The products of *in vitro* selection, aptamers are short nucleic acids (DNA or RNA) that bind their targets with high affinity and specificity. Aptamers can be expressed on demand from transgenes and are intrinsically amenable to recognition by nucleic acid-binding proteins such as TFs. In this study, we review several natural prokaryotic and eukaryotic examples of RNAs that modulate the activity of TFs. These examples include 5S RNA, 6S RNA, 7SK, hepatitis delta virus-RNA (HDV-RNA), neuron restrictive silencer element (NRSE)-RNA, growth arrest-specific 5 (Gas5), steroid receptor RNA activator (SRA), trophoblast STAT utron (TSU), the 3′ untranslated region of *caudal* mRNA, and heat shock RNA-1 (HSR1). We then review examples of unnatural RNA aptamers selected to inhibit TFs nuclear factor-kappaB (NF-κB), TATA-binding protein (TBP), heat shock factor 1 (HSF1), and runt-related transcription factor 1 (RUNX1). The field of RNA aptamers for DNA-binding proteins continues to show promise.

## Introduction

**FIG. 1.**
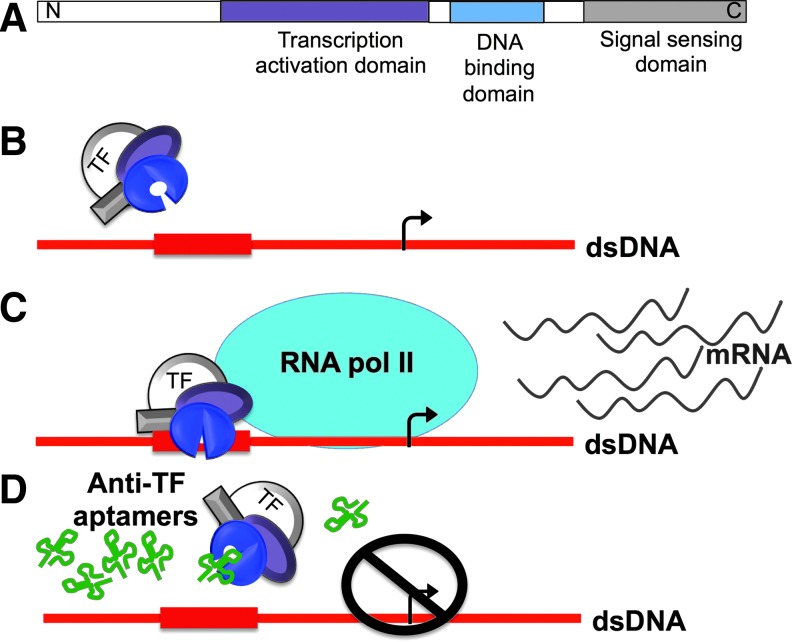
Example of transcription factor (TF) modular structure and proposed mechanism of anti-TF aptamers. **(A)** Schematic illustration of an example TF showing separate structural modules with different functions: transcription activation domain, DNA-binding domain, and signal-sensing domain. **(B)** Potential mechanism of anti-TF aptamers. TFs are activated and bind to promoter and enhancer consensus sequences. **(C)** Upon DNA binding TFs promote and regulate chromatin modification and recruitment of RNA polymerase. **(D)** Aptamers with high specificity and affinity against a TF might competitively bind the target and inhibit binding of TF to dsDNA, resulting in inhibition of gene expression.

Regulation of gene expression is crucial for the development and survival of cells, resulting in exquisite control of the function and development of living organisms. Gene expression is regulated at many stages, but a dominant role is played by control of transcription initiation. Crucial in this process are sequence-specific DNA-binding proteins termed transcription factors (TFs). Possessing modular structures often including a DNA-binding domain and a transcriptional activation or repression domain, some TFs also contain signal-sensing domains (Fig. 1A) [1,2]. TFs can regulate transcription either positively or negatively [3,4]. Because of their specificity and role in controlling gene expression, TFs make compelling targets for therapeutic manipulation to control genes that are either deregulated due to derangement of signaling cascades, or due to the deregulation of the TF itself. While it is commonly recognized that TFs are attractive therapeutic targets for the next generation of drugs, there has been little progress toward this goal [5,6].

Currently most marketed drugs are small molecules, less likely to compete with large charged molecular surfaces such as those involved in TF binding to DNA. In this study we review the intriguing cases of natural and selected RNA aptamers that bind and competitively inhibit TFs.

Aptamers are short RNA or DNA sequences that fold into complex three-dimensional structures and bind to their targets with high affinity and specificity. They are typically the product of the *in vitro* technique termed SELEX (systematic evolution of ligands by exponential enrichment) [7,8]. Several considerations make aptamers intriguing tools for TF inhibition. Target affinity can be comparable to antibodies (nanomolar to picomolar range), moderate molecular mass allows access to smaller biological compartments, targeting is flexible, the agents appear to be nonimmunogenic, and high specificity can be achieved. For example, an aptamer to growth factor fibroblast growth factor-2 (FGF-2) reportedly binds 20,000-fold more tightly to its target than to closely related FGF homologs [9], and synthetic aptamers can be modified to increase bioavailability while preserving ease of preparation and lack of toxicity [10,11]. Recent advances in high-throughput technology have improved aptamer selection [12–15]. While nucleic acid aptamers face the obvious challenge of cell penetration, RNA aptamers have the unique advantage that they can be encoded in transgenes for endogenous expression after gene delivery.

Anti-TF aptamers have been conceived as therapeutic agents, either to inhibit the expression of genes that are transactivated by the target TF, or to activate genes that are transcriptionally repressed by the target TF. In principle, appropriate RNA aptamers can be selected for binding to the DNA-binding domain of a target TF, blocking it from binding to its double-stranded DNA target site and thereby competitively inhibiting its activity (Fig. 1B–D). TF inhibition by double-stranded DNA copies of the TF-binding site represents the simplest implementation of this concept. Such an approach was applied to E2F-1 with the goal of preventing a common cardiovascular disorder [16,17]. In this study, we focus instead on the intriguing concept of RNA aptamers against DNA-binding TFs where the opportunity for therapeutic expression from transgenes can be considered and the fascinating problem of RNA mimicry of DNA comes into play. We review both natural and *in vitro*-selected anti-TF RNA aptamers.

## Natural Occurring Anti-TF Aptamers

A fascinating class of RNAs appear to function in the modulation of protein activity by mimicking the structures of other DNAs or RNAs. These natural RNAs act in a manner reminiscent of the proposed TF inhibitors described above (Table 1) [18].

**Table 1.** Natural RNAs That Regulate Transcription Factors

*RNA*	*TF target*	*Regulation*
5S RNA	TFIIIA	Its own transcription
6S RNA	σ^70^-RNAp	Wide-range regulation of transcription of several genes
3′ UTR of cad	Bicoid	Expression of caudal
TSU	STAT1	Reduces nuclear translocation and suppression of MHC genes
NRSE-RNA	NRSF/REST	Inhibits REST from binding to dsDNA and acting as a repressor
HSR1	HSF1	Stimulates heat shock response by stabilizing HSF1
HDV	RNAP II	Regulates its own transcription
7SK	HMGA1	Inhibits HMGA1 binding to dsDNA
ncRNA anti-p53	p53	Fine tunes p53 response
Gas5	GR	Reduces GR response
SRA	SRA	Activates SRA response by acting as a scaffold

Gas5, growth arrest-specific 5; GR, glucocorticoid receptor; HDV, hepatitis delta virus; HMGA1, high mobility group protein A1; HSF1, heat shock transcription factor 1; HSR1, heat shock RNA-1; MHC, major histocompatibility complex; NRSE, neuron restrictive silencer element; NRSF, neuron-restrictive silencer factor; REST, RE1-silencing transcription factor; RNAP II, RNA polymerase II; SRA, steroid receptor RNA activator; STAT1, signal transducers and activators of transcription; TF, transcription factor; TSU, trophoblast STAT utron; UTR, untranslated region.

### 5S rRNA

**FIG. 2.**
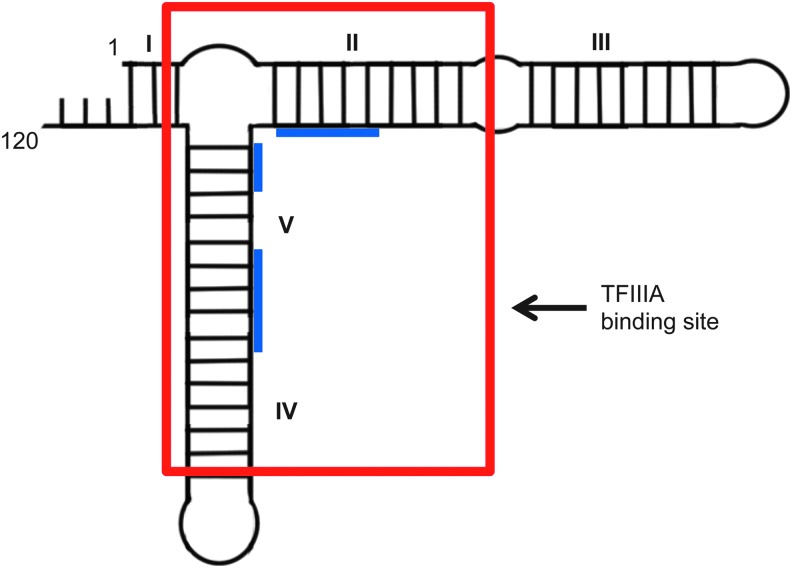
*Xenopus laevis* 5S RNA. Schematic secondary structure of *X. laevis* oocyte 5S rRNA. Helices and loops are *numbered*. The putative TFIIIA-binding site is *boxed* in *red* and physical interaction regions with zinc fingers are shown in *blue*. Adapted from Romby *et al.* [162].

5S rRNA is a universal component of the large ribosomal subunit. Although it is essential for the activity of the ribosome, its precise role remains elusive. Transcription of 5S rRNA during oogenesis in *Xenopus laevis* is controlled by TFIIIA, a positive regulator that binds to an internal control region of the 5S rRNA gene [19,20]. TFIIIA is a zinc metalloprotein [21] composed of nine classical cys_2_-his_2_ zinc fingers arranged consecutively [22]. In the 1980s it was discovered that TFIIIA possesses the remarkable ability to bind to the transcript of the gene it controls, 5S rRNA, forming a storage ribonucleoprotein particle (7S RNAP) (Fig. 2). These particles accumulate to massive levels in the oocyte before ribosome assembly [19,23–26]. Thus, although TFIIIA is cataloged as a DNA-binding protein, it has the unusual ability to interact specifically with both double-stranded DNA and with RNA.

The competitive nature of DNA versus RNA binding initially suggested that the same binding domain of TFIIIA interacts with both DNA and its RNA transcript. However, biochemical analysis and subsequent high-resolution structure determination elucidated the more surprising and complex recognition mechanism for these nucleic acids. *In vitro* analysis of a series of TFIIIA zinc finger deletions revealed that fingers 1–3 (numbered from N- to C-terminus) contributed most to the interaction with DNA, while fingers 4–6 where largely responsible for RNA binding [27].

These biochemical data were subsequently supported by high-resolution structural studies using X-ray crystallography [28] and nuclear magnetic resonance (NMR) [29]. Different sets of zinc fingers indeed dominate the different interactions between TFIIIA and its cognate DNA and RNA partners through induced fit interactions [29]. It is important to note that when TFIIIA binds 5S rRNA, the protein can no longer bind DNA, despite the involvement of different fingers. Thus, although 5S RNA is not acting as a perfect mimic of the TFIIIA target DNA, it effects competitive inhibition by a mechanism comparable to that proposed above.

### 6S RNA

Another striking natural example of an RNA that acts as a competitive inhibitor of a DNA-binding protein is provided by prokaryotic 6S RNA. This small RNA is important for the bacterial stress response upon nutrient deprivation [30]. Detected initially because of its high abundance in *Escherichia coli* [31], this RNA was subsequently shown to inhibit normal transcription by binding directly to the housekeeping holoenzyme form of RNA polymerase (σ^70^-RNAp), preventing its binding to gene promoters. Remarkably, 6S RNA forms a stable, long-lived complex with σ^70^-RNAp, but not with free polymerase or RNA polymerases containing alternative σ subunits [32–34].

6S RNA controls a large number of genes by downregulating the transcription of most σ^70^-dependent promoters. 6S RNA accumulates to high levels during late stationary phase and binds efficiently to σ^70^-RNAp. During this time, most 6S RNA is bound to the polymerase. This explains downregulation of transcription at σ^70^-dependent promoters [35–37]. During the exponential phase, in contrast, most σ^70^-RNAp is bound to DNA. 6S RNA inhibition of specific promoters leads to an altered program of gene expression, apparently adapting to the nutrient stress of stationary phase. When cells are moved to rich media, nucleotide triphosphate (NTP) concentrations rise and 6S RNA apparently becomes a template for RNA polymerase, generating a small RNA product (pRNA), and resulting in the release and degradation of 6S RNA and recycling RNA polymerase.

**FIG. 3.**
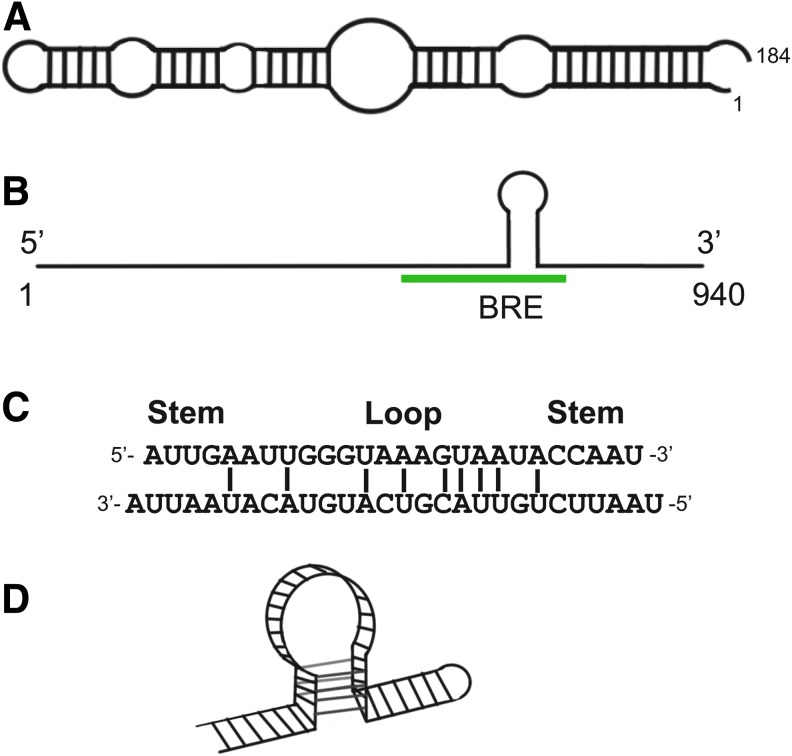
Natural anti-TF RNA aptamers. **(A)** *Escherichia coli* 6S RNA. Schematic of 6S RNA secondary structure, adapted from Wassarman [33]. **(B)** Schematic of caudal (*cad*) mRNA showing 940-nucleotide transcript. Highlighted in *green* is the region found to interact with bicoid protein, termed the bcd recognition element (BRE). The hairpin depicted in this region is a hypothetical secondary structure proposed for the interaction with bcd. **(C)** Hypothetical trophoblast STAT utron (TSU) model involving base pairing of promoter-like motifs 1 and 8 in TSU. **(D)** Synthetic TSU model RNA based on GAS motifs in loop–loop bent helical structures. **C** and **D** adapted from Peyman [47].

Computer predictions and structural mapping have shown that 6S RNA is largely double-stranded with a single-stranded central bulge (Fig. 3A). This conserved secondary structure is required for 6S RNA interactions with σ^70^-RNAp [32]. The resemblance of the 6S RNA secondary structure to the conformation of promoter DNA within an open complex led to speculation that the 6S RNA might interact with RNA polymerase as a mimic of promoter DNA [34]. This speculation was later confirmed by biochemical studies where 6S RNA was found to be engaged at the RNA polymerase active site, as it actually can serve as a functional template to generate pRNAs [38]. Thus, when 6S RNA is bound to the active site of the RNA polymerase in the presence of low NTP concentrations, the RNA inhibits transcription by a decoy function, sequestering RNA polymerase from binding DNA promoters.

### 3′ Untranslated region of caudal

Arthropods, such as the fruit fly *Drosophila melanogaster,* are composed of body segments. During early embryonic development, the segments of the embryo adopt their own identities. The primary determinant of anterior pattern in the embryo is the graded expression of *bicoid* (*bcd*), a gene encoding a homeodomain TF. Homeodomain proteins are transcriptional regulators that specify the body plan by controlling transcription of their target genes. *Bcd* mRNA is tightly localized to the anterior pole of the egg [39,40]. After fertilization, this mRNA is translated giving rise to an anterior-to-posterior gradient of bcd protein that then activates the transcription of target genes at distinct concentration thresholds [39–43]. Transcription activation by bcd is mediated by direct binding of the homeodomain to DNA targets. Shortly after the bcd gradient is established, a second homeodomain protein, caudal (cad), accumulates in an opposing posterior-to-anterior gradient under bcd control.

The regulation of cad by bcd is necessary for proper patterning. Inappropriate expression of *cad* causes deletions of head and thoracic segmentation [44]. Unexpectedly, it was found that regulation of cad by bcd occurs at the level of translation. The bcd homeodomain acts on the 3′ untranslated region (UTR) of the cad message [45,46]. Just as in the examples presented above, bcd binding to the 3′ UTR of cad mRNA (Fig. 3B) excludes bcd from binding to DNA. In this case, bcd simultaneously acts as a translational repressor of cad. Thus, it is a remarkable feature that a single protein executes both transcriptional and translational control. The detailed mechanism by which bcd binds both DNA and RNA remains speculative. However, the proposed model is that the bcd DNA recognition domain contacts RNA in a similar manner to the basic domain of another protein that recognizes RNA, the Rev protein of human immunodeficiency virus (HIV).

Other portions of the bcd homeodomain, such as the N-terminal arm, are also rich in arginine residues and may stabilize this interaction by making additional contacts with the RNA. The premise that the same protein residues involved in DNA recognition may also be involved in binding RNA is supported by the observation that amino acid substitutions in the bcd DNA recognition α-helix can block the translational repression of cad [45].

### Trophoblast STAT utron

A repressor RNA termed trophoblast STAT utron (TSU) has been reported to bind TF signal transducers and activators of transcription (STAT1) [47], a sequence-specific TF involved in immune function and development. Complex formation between STAT1 and TSU appeared to reduce STAT1 nuclear translocation and repress major histocompatibility complex (MHC) class II expression in the early embryo [47]. Sequence-specific binding was suggested by results of experiments with RNA constructs carrying 80 nucleotides of the TSU transcript, including two predicted stem-loops and a central hairpin. Electrophoretic gel mobility shift studies showed that the loop–loop structures with the partially complementary motif 1 (5′GUAAAGUAA3′) and motif 8 (5′UUACGUCAU3′) formed complexes with STAT1, while controls did not (Fig. 3C, D). It has been proposed that STAT1 binds TSU using motifs similar to the specific STAT DNA-binding site, suggesting that the protein employs a similar mechanism in binding both DNA and RNA. Subsequent characterization of the TSU system has not been reported.

### Neuron-restrictive silencer element-RNA (RE1-RNA)

**FIG. 4.**
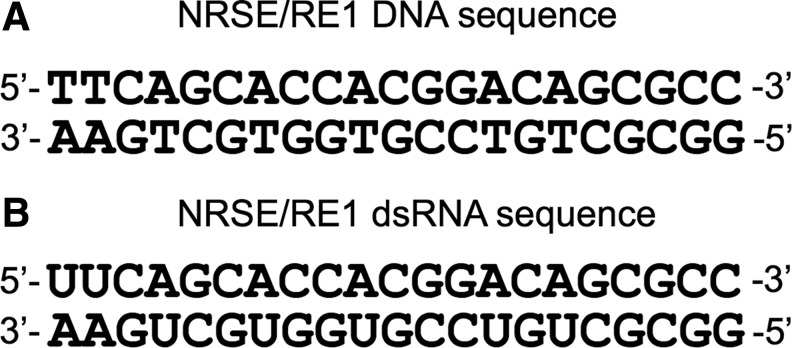
Schematic diagram of neuron-restrictive silencer factor (NRSF)/RE1-silencing transcription factor (REST) sequence specificity. **(A)** Neuron-restrictive silencer element (NRSE/RE1) element found in dsDNA. **(B)** Identical NRSE/RE1 sequence found in NRSE-RNA.

RNAs extracted from adult hippocampal neural stem cells included a double-stranded RNA containing a sequence corresponding to a 21 base pair DNA element termed neuron-restrictive silencer element (NRSE), also known as RE1 (Fig. 4) [48]. Further experiments suggested that this RNA interacts with the neuron-restrictive silencer factor (NRSF) [49], also known as RE1-silencing transcription factor (REST) [50].

NRSF/REST is a Krüppel family of zinc finger transcription repressor expressed at high levels in most non-neuronal cells and in undifferentiated neuronal progenitors. However, its expression is low in mature neurons [49–52]. REST binds the conserved NRSE/RE1 DNA element, where it acts as a modular scaffold for the assembly of diverse macromolecular complexes blocking transcription. REST represses hundreds of neuronal genes, including those encoding ion channels, neurotrophins, neurotransmitters, synaptic vesicle proteins, and a broad range of factors involved in neurite growth, axonal guidance, and transport [53,54].

It was shown that the noncoding NRSE-RNA binds transcription factor REST during a defined period of neuronal differentiation and effects changes in REST-dependent gene expression, thus modulating the function of REST between activation and repression of neurogenesis. Electrophoretic gel mobility shift assays revealed that the affinity of NRSF/REST for NRSE-RNA was much higher than its affinity for NRSE double-stranded DNA. Moreover, the expression of NRSE-RNA was shown to be necessary and sufficient to direct multipotent neuronal stem cells toward a neuronal fate, suggesting that this RNA can function as an endogenous inducer of neuronal differentiation [48]. Based on immunoprecipitation experiments and mutation analysis, it was inferred that there is a physical interaction between NRSE-RNA and REST protein [48].

A simple model was proposed in which NRSE-RNA-dependent gene activation is induced through the physical interaction of the RNA as a competitive decoy for REST, releasing the genome from repression. Although structural studies are lacking, it has been suggested that the interaction of NRSE-RNA with NRSF/REST involves one of the eight zinc fingers of the protein [48]. Zinc finger-containing proteins have the potential to bind either RNA or double-stranded DNA, as described above in the case of TFIIIA. Although NRSE-RNA competes for binding against the double-stranded DNA element RE1, it is possible that NRSF/REST binds through different zinc fingers than those involved in DNA binding.

It is important to note that *in vivo* studies from two different groups [48,55] showed stimulatory effects attributed to the sequestration of NRSF/REST when using vectors intended to express NRSE-RNA from NRSE/RE1 DNA sequences. However, Kuwabara *et al.* pointed out that these effects were probably due to sequestration of NRSF/REST by the plasmid DNA, and not by expressed NRSE-RNA. These false positive results point to the potential advantage of anti-REST RNA aptamers. It has previously been shown that RNA aptamers can bind DNA-binding proteins without encoding the same cognate DNA recognition sequences, avoiding the situation where both a DNA template and its RNA product both interact with a protein.

A broad range of neurological diseases, including glioma, stroke, and neurodegeneratation (including Huntington's disease), are characterized by deregulation of REST. In fact, most of these disorders are characterized by increased REST activity, leading to transcriptional repression. Thus, REST represents a candidate for therapeutic TF inhibition [56–58] for treatment of these diseases [59].

### Heat shock RNA-1

Heat shock RNA-1 (HSR1) is a noncoding RNA that stimulates the mammalian heat shock response. This response is a major cellular defense mechanism after cellular stress. During heat shock, rapid and substantial changes occur in the pattern of gene expression. HSR1 activates the heat shock transcription factor 1 (HSF1), which is essential for the induction of expression of heat shock proteins (HSPs) and other cytoprotective proteins [60]. HSR1 was identified in a screen seeking putative auxiliary factors involved in the activation of HSF1. A complex of HSF1 and elongation factor (eEF1A) coimmunoprecipitated from cell lysates. Initial *in vitro* studies seeking to activate HSF1 with eEF1A isolated from cells were unsuccessful.

**FIG. 5.**
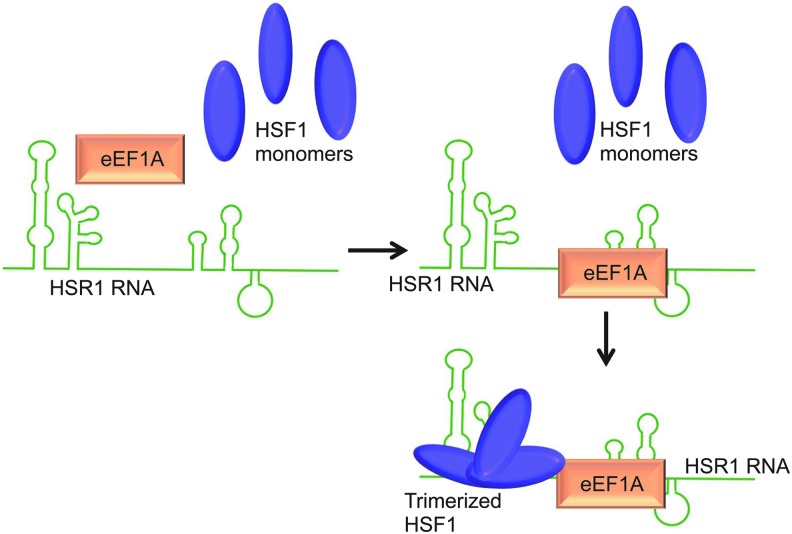
Heat shock RNA-1 (HSR1) mechanism of action. The HSR1-eEF1A complex is proposed to facilitate and stabilize the trimerization of HSF, which binds at the 5′ terminus of HSR1. HSR1-eEF1A complexes are necessary for activation of heat shock transcription factor 1 (HSF1).

Moreover, HSF1 binding to DNA was strongly inhibited *in vitro* by preincubation of the cell lysate with RNase A, which strongly pointed to the involvement of an RNA. HSR1 is ∼600 nucleotides in length, lacks a poly(A) tail, and acts together with eEF1A to activate transcription factor HSF1 [61]. Two domains near the 5′ terminus of HSR1 are essential for activation of HSF1 (Fig. 5). *In vivo* studies using vectors expressing siRNA against different domains of HSR1 supported previous findings: heat shock induction of HSF1 DNA-binding activity was severely impaired in siRNA-treated cells and not in controls [61]. It was hypothesized that the noncoding HSR1 RNA forms a complex with eEF1A that then binds and facilitates the assembly and stability of HSF1. HSR1 provides an example of a noncoding RNA that may be essential for the proper function of a TF. This makes HSR1 an interesting pharmacological target for various conditions associated with HSF1 activation, such as inflammation, ischemia/reperfusion, and cancer [61,62].

### Hepatitis delta virus

**FIG. 6.**
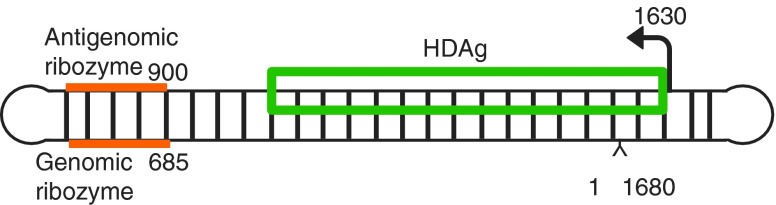
Schematic depiction of hepatitis delta virus genome. The delta ribozyme motifs (dRz, in *orange*) and their respective cleavage sites are indicated. The promoter on the genomic strand (indicated by the *arrow*) and the region decoding the HDAg mRNA is *boxed* (in *green*). This scheme was adapted from Kuo *et al.* [163].

Hepatitis delta virus (HDV) provides a particularly remarkable example of double-stranded DNA mimicry by RNA. HDV is the smallest known human RNA pathogen. It is a defective virus that requires the hepatitis B virus envelop proteins for encapsidation and propagation [63–65]. This RNA folds on itself to form a rod-like structure that can be divided into two domains (Fig. 6) [64,66,67]. The left terminal domain includes both genomic and antigenomic self-cleaving RNA motifs. The right terminal domain contains a single open reading frame encoding two viral proteins, the small HDAg (HDAg-S) and the large HDAg (HDAg-L). Although these proteins are almost identical, each plays a distinct role. HDAg-S is essential for HDV replication, while the HDAg-L is necessary for virion assembly [66,68,69]. Replication of the HDV RNA apparently takes place in the nucleus of infected cells using a symmetrical rolling cycle mechanism, in which replication of the infectious circular RNA monomer produces linear, multimeric strands that are subsequently cleaved by endogenous ribozymes and ligated, yielding antigenomic circular monomers. These RNAs are then used as templates. The same three steps are repeated to generate genomic RNA progeny [70,71].

As HDV does not encode its own RNA-dependent RNA polymerase (RNApol), a host DNA-dependent RNA polymerases (RNApol) must somehow be involved in the replication and transcription of HDV RNAs. In fact, RNA polymerase II (RNApol II) was reported to interact with HDV-derived RNAs at sites located within the terminal stem-loop domains [72,73]. Mutagenesis near the terminal loops of the rod affected both HDV accumulation in cells and RNApol II binding *in vitro* [73–75]. Furthermore, an RNA fragment derived from the right terminal stem-loop region of genomic HDV-RNA, including the site of HDAg mRNA transcription has been shown in several experiments to serve as a template for *in vitro* transcription. Using RNA affinity chromatography it was established that an RNAP II preinitiation complex forms on this promoter RNA, analogous to what is observed on double-stranded DNA promoters during transcription [75,76]. The crystal structure of purified RNApol II engaged in transcription of an HDV-RNA was solved. When superimposed with the structure of RNApol II engaging a DNA template, it was evident that both nucleic acids occupy the same site. These striking findings suggest that RNApol II recognizes the HDV RNA and normal DNA templates in a similar way [77]. RNApol has been found to engage RNA as template in other cases, including the peach latent mosaic viroid RNA genome [78,79] and endogenous bacterial 6S RNA [38]. Thus, certain RNA viruses have exploited the ability of special RNA structures to mimic double-stranded DNA with sufficient accuracy that they can recruit a host DNA-dependent RNA polymerase as their replicase.

### 7SK

The nuclear noncoding 7SK RNA is a highly abundant RNA found in eukaryotic cells. 7SK RNA is believed to negatively regulate transcription elongation by inactivating the positive transcription elongation factor b (P-TEFb) [80–82]. In the past decade it has been reported that 7SK interacts with the high mobility group protein A1 (HMGA1) [83]. HMGA1 proteins are highly expressed during development, apparently influencing cell proliferation, embryonic cell growth, and cell differentiation [84–88].

HMGA proteins have three AT-hook DNA-binding motifs that allow them to preferentially bind in the minor groove of AT-rich, B-form DNA sequences [89]. HMGA1 proteins may facilitate gene transcription by strongly altering DNA structure, resulting in a more open chromatin state. These proteins also physically interact with many different TFs, orchestrating their assembly at promoter and enhancer regions [90–92]. The interaction of 7SK RNA and HMGA1 is reported to be highly specific. 7SK RNA interacts with the N-terminal domain of HMGA1, binding to an AT-hook motif through the second major hairpin of the RNA (loop 2) [83].

**FIG. 7.**
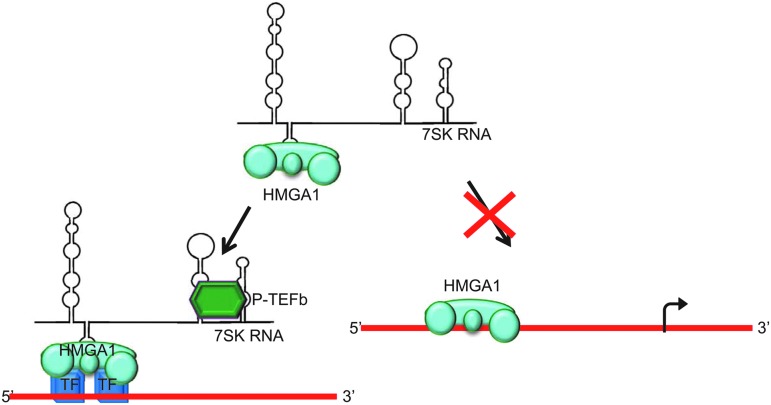
Cellular function of 7SK-HMGA1 complex. 7SK RNA acts as a negative regulator of high mobility group protein A1 (HMGA1) DNA-binding, subsequently regulating HMGA1 target gene expression. Adapted from Benecke and Eilebrecht [93].

7SK RNA was shown to compete with DNA for HMGA1 binding, thus functioning as a competitive transcription regulator (Fig. 7) [83,93]. Overexpression of the 7SK loop 2 structure as a chimera with the Epstein–Barr virus EBER2 RNA was reported to alter gene expression in a manner similar to knockdown of HMGA1, supporting the apparent regulatory function of 7SK on HMGA1 [83].

Mysteriously, HMGA1 proteins are frequently overexpressed in tumor cells, with HMGA1 expression levels often correlated with tumor malignancy. This has suggested that HMGA1 proteins might be targeted therapeutically [94,95]. The natural role of 7SK RNA as an HMGA1 antagonist suggests that this RNA might be manipulated for such therapeutic purposes. Finally, it has also been reported that HMGA1 proteins interact with other RNA molecules, including the transactivating response element in the nascent transcript of HIV-1 [96,97]. This observation emphasizes the promiscuity of HMGA1 as a dual DNA/RNA-binding protein, a recognized theme in biology [98].

### p53

**FIG. 8.**
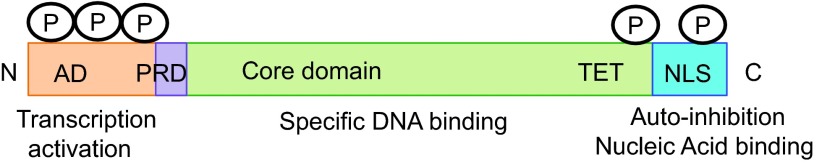
p53 protein biochemistry and putative regulatory interactions. p53 domains. AD, activation domain; PRD, proline-rich SH3-binding domain; TET, tetramerization domain; NLS, nuclear localization sequence; P, phosphorylation sites. Adapted from Cassiday and Maher [98].

The p53 tumor suppressor protein plays a prominent role in cell growth, DNA repair, cell cycle arrest, and apoptosis. Mutation of p53 is among the most prevalent abnormalities in human cancer [99]. Although p53 is classified as a sequence-specific DNA-binding TF, it has also been reported to bind to RNA. Unlike many of the cases described above, interaction of p53 with RNA and DNA appears to be through two distinct binding domains [100]. The p53 core contains the DNA-binding domain, which recognizes the p53 consensus sequence in promoters of target genes (Fig. 8). RNA and single-stranded DNA, however, are recognized by the cationic C-terminus, which is involved in the regulation of p53 activity [100]. Posttranslational modification of the p53 C-terminus activates sequence-specific DNA binding by the DNA-binding domain, apparently by relieving autoinhibition [101]. Nucleic acid binding to the p53 C-terminus, therefore, has the potential to alter p53 function [102].

The physiological significance of RNA-p53 interactions remains controversial. It has been shown that the unmodified cationic p53 C-terminus binds RNA strongly and with little sequence specificity both *in vitro* and in the yeast three-hybrid system [103]. In contrast, physiologically relevant posttranslational modification of the p53 C-terminal domain abrogates this nucleic acid binding [104,105]. Because different p53 isoforms are expressed under specific cell conditions, and posttranslational modifications are variable, it remains possible that RNA binding at the p53 C-terminus could have a rare physiological role. Emerging research into the function of long noncoding RNAs also suggests that these RNAs could play roles in fine-tuning p53 function [106].

## RNAs That Regulate Activity of Nuclear Receptors

Nuclear receptor (NR) TFs respond to small molecule metabolites and fat-soluble compounds to regulate gene expression. They differ from other receptors in their unique ability to directly control gene expression through binding to genomic DNA, thus being classified as TFs [107,108]. NRs allow organisms to respond correctly to their environment by coordinating multicellular metabolism, development, reproduction, and homeostasis across diverse tissues [109]. NRs possess a conserved architecture and signaling mechanism. The DNA-binding domain contains two zinc fingers near the N-terminus, which contact the double-stranded DNA helix and form a dimerization interface. The ligand-binding domain resides at the C-terminus and engages cognate hormones as well as coactivators and corepressor complexes. NRs are classified based on their mechanism of action or homology. Class 1 receptors, such as the classical steroid receptors [eg, estrogen, androgen, and glucocorticoid receptors (GRs)], are activated by ligands, while the class 2 receptors [eg, peroxisome proliferator-activated receptors (PPARS), vitamin D receptor, and thyroid receptor] function as transcriptional activators in the presence of ligand, but as repressors in the absence of ligand [109,110].

In the past decade, an increasing number of noncoding RNAs with regulatory functions have been reported [111]. Two of these RNAs have been proposed as regulators of NRs, the growth arrest-specific 5 (Gas5) RNA, and the steroid receptor RNA activator (SRA).

### Growth arrest-specific 5

Gas5 RNA was so named because it has been observed to accumulate in growth-arrested cells [112]. Surprisingly, Gas5 RNA has been reported to interact with the DNA-binding domain of the ligand-activated GR and suppress GR-induced transcription of endogenous glucocorticoid-responsive genes by competitive inhibition of GR binding to target glucocorticoid response elements (GREs) [113]. Glucocorticoids, a class of steroid hormones, serve as intracellular mediators that link systemic physiology to cellular activities [114].

Gas5 RNA apparently functions as a starvation-linked or growth arrest-linked riborepressor of GR. Gas5 RNA was identified by a LexA-based yeast two-hybrid screen with the Jurkat cell complementary DNA (cDNA) library, using the GR DNA binding domain (DBD) as bait. Increased association of GR and Gas5 RNA was observed by coimmunoprecipitation when HeLa cells were treated with dexamethasone, a GR agonist. Evidence of Gas5 RNA association with the GR DNA-binding domain came from results of experiments in which a GR chimera, G-gal-G, in which the DBD was replaced with that of the bacterial transcription factor GAL4. This chimera showed no interaction with Gas5 RNA [113]. Gas5 RNA is detected in the cytoplasm, but is more prominent in the nucleus [113,115].

**FIG. 9.**
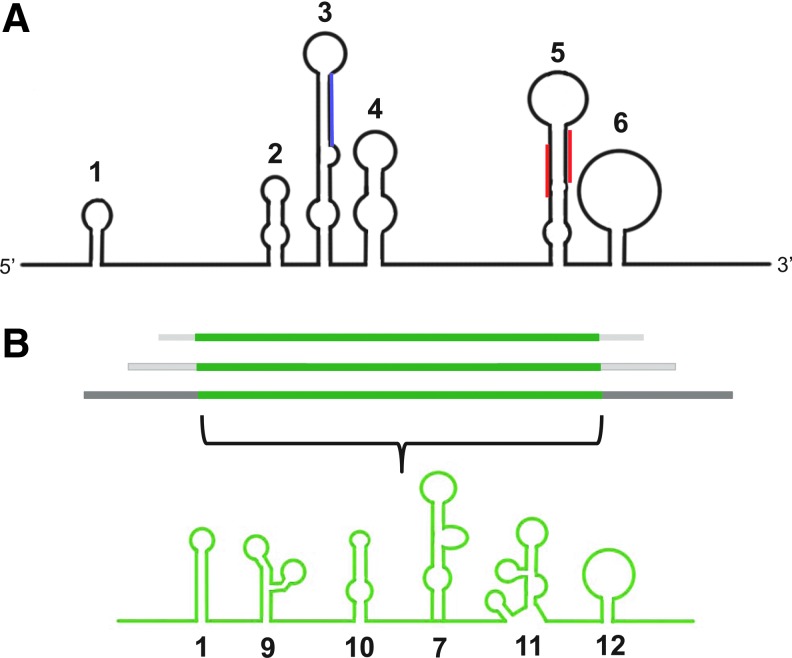
RNAs that regulate nuclear receptors. **(A)** Schematic secondary structure of growth arrest-specific 5 (Gas5) nucleotides 400–598. Gas5 (400–598) consists of six hairpin structures. Hairpin 5 is proposed to contain two glucocorticoid response element (GRE) sequences at nucleotides 539–544 and 553–559 (shown in *red*), which form a double-stranded hairpin structure that may mimic a GRE in dsDNA. Gas5 hairpin 3 is proposed to contain an MRE mimic (highlighted in *purple* at nucleotides 540–554). These residues are conserved among the consensus DNA GREs and are thought to be critical for interaction with residues of the glucocorticoid receptor DNA binding domain. Adapted from Kino [113]. **(B)** Three SRA1 complementary DNAs (cDNAs) sharing a central core region, but different in their 5′ and 3′ extremities and below a schematic representation of the proposed steroid receptor RNA activator (SRA) RNA secondary structures and assigned motifs. Adapted from Refs. [164,165].

Studies thus suggest that Gas5 RNA accumulates in growth-arrested cells in response to serum withdrawal, acting as a negative regulator of GR-induced transcription. Gas5 RNA is encoded by the Gas5 gene, which produces two mature, spliced forms of Gas5 RNA. The full-length Gas5b RNA is 630 nucleotides long and forms hairpin structures (Fig. 9A), apparently interacting with GR through a particular 3′ sequence. Full-length RNA and fragments containing nucleotides 400 to 598 reportedly bound GR in response to suppressed GR-induced transcriptional activity [113]. This region of Gas5 RNA contains two GRE-like sequences predicted to be base-paired within a hairpin structure (containing a G540 in the 5′ strand and a C554 in the 3′ strand). These RNA sequences resemble the consensus DNA GRE sequence and define affinity to the GR DNA-binding domain [113,116]. These Gas5 RNA GRE-like sequences were found to be necessary for binding to GR, with equilibrium dissociation constant estimates of ∼30 nM, tighter than those reported for GR binding to its cognate DNA (∼60 nM) [117]. Gas5 is thus mechanistically reminiscent of the bacterial 6S RNA, which has been documented to bind RNA polymerase and inhibit transcription by mimicking the open promoter to which RNA polymerase binds [118,119]. Gas5 is, therefore, another putative competitive inhibitor of a TF, apparently acting through RNA mimicry of double-stranded DNA. Particularly mysterious in this case is how the GRE-like sequences are recognized by GR in the context of the A-form helical structure of duplex RNA.

### Steroid receptor RNA activator

The steroid receptor coactivator 1 (SRC-1), as well as an RNA that increased the transcription activation of steroid receptors, were found when searching for coactivators of NRs using a yeast two-hybrid screening assay normally used to identify protein–protein interactions [120]. The unexpected RNA activator, termed SRA, appears to be transcriptional coactivator that acts in a manner selective for the amino-terminal transcription activation function (AF-1) of steroid receptors. It is expressed as multiple isoforms in a cell-specific manner [120]. The apparent transcriptional regulatory activity of SRA has been confirmed by overexpression in mammalian cells, where it is reported to enhance steroid receptor-mediated transactivation without significantly enhancing the levels of basal transcription from other promoters.

Treatment of cells with antisense oligodeoxyribonucleotides against SRA reportedly induced ∼70% reduction of the steroid-dependent transcription [120]. SRA differs from eukaryotic transcriptional activators in its ability to function as an RNA transcript that selectively regulates the activity of a family of transcriptional activators, existing in distinct ribonucleoprotein complexes. Thus SRA is apparently expressed in steroid target tissues and functions as a component of a large multiprotein complex to selectively enhance transcriptional activation in this context. SRA has several isoforms, all of them containing an identical core region of 687 nucleotides, but diverging in length and sequence in 5′ and 3′ regions. It has been proposed that SRA can serve as an organizing platform upon which relevant molecular components are assembled (Fig. 9B) [121]. In this regard, SRA is more a natural organizer of protein–protein interactions on DNA than an inhibitor of DNA–protein interactions.

## Synthetic Anti-TF Aptamers

Natural examples of RNAs that function as mimics of DNA and inhibit TFs have inspired several research groups to use random RNA libraries and *in vitro* selection to seek engineered competitive TF inhibitors of this type. This work has led to the description of several unnatural RNA aptamers with potential for therapeutic control of gene expression (Table 2).

**Table 2.** Selected RNAs That Regulate Transcription Factors

*RNA aptamer*	*Target*	*Potential application*
Anti-NF-κB	Homodimer p50, heterodimer p50/p65	Inhibitors of NF-κB
Anti-TBP	TBP, TBP•TATA	Tool to study protein–protein interactions and inhibition
Anti-HSF1	HSF1	Tool to understand transcriptional mechanisms and inhibitors
Anti-RUNX1	RHD-CBFβ, Runt	Inhibitors

CBF, core-binding factor; NF-κB, nuclear factor-kappaB; RHD, runt homology domain; RUNX1, runt-related transcription factor 1; TBP, TATA-binding protein.

### Anti-nuclear factor-kappaB aptamers

*In vitro* selection was used to identify a high-affinity RNA aptamer specific for p50-containing forms of TF nuclear factor-kappaB (NF-κB). This TF is an important activator of genes involved in diverse biological activities, including cell proliferation, cell growth, resistance to apoptosis, and immune functions, such as inflammation and the synthesis of chemokines, interferons, MHC proteins, growth factors, and cell adhesion molecules [122]. NF-κB also plays a key role in the expression of HIV-1 genes after lymphocyte activation [123]. Inactive NF-κB protein is localized in the cytoplasm bound to inhibitor of kappaB (I-κB). Upon activation by I-κB phosphorylation, I-κB is ubiquitinated and degraded, releasing NF-κB to be translocated to the nucleus for activation of target gene expression.

With aims of generating a potential candidate for inhibition of NF-κB-dependent gene activation, a high-affinity 31-nucleotide RNA hairpin aptamer was identified as a subdomain of a larger RNA developed through *in vitro* selection [124]. *In vitro* and *in vivo* selections were then used to improve the affinity of anti-NF-κB for p50_2_ and heterodimer p50/p65 [124–126]. Other selections have identified different RNA aptamers against p65 NF-κB subunits [127,128].

**FIG. 10.**
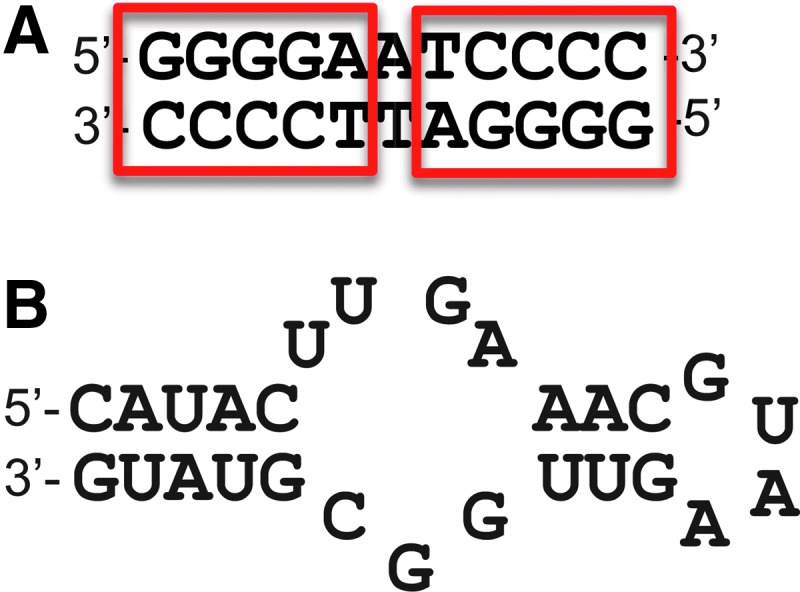
Nuclear factor-kappaB (NF-κB) interacting nucleic acids. **(A)** Example κB DNA sequence from the MHC1 class I gene promoter (*red boxes* illustrate DNA half-sites). **(B)** Sequence and secondary structure of the *in vitro* selected anti-NF-κB RNA aptamer that binds the NF-κB p50 subunit. Adapted from Ghosh *et al.* [166]. MHC1, major histocompatibility complex class 1.

Anti-p50 RNA aptamers were found to bind with nanomolar affinity (*K*_d_ ∼ 1 nM) in a 1:2 RNA-to-p50 ratio in solution [129]. The RNA was found competent to strongly bind NF-κB p50 protein *in vivo* using the yeast three-hybrid system [125,126]. Interestingly, when the 2.45 Å resolution cocrystal structure of the NF-κB p50 Rel homology domain homodimer bound to a 29-nuclotide form of the anti-NF-κB RNA aptamer was solved, it was revealed that one RNA molecule binds identically to each of the p50 monomers of the homodimer, forming a RNA_2_:p50_2_ complex [130]. Each RNA hairpin is folded into an irregular helix characterized by a series of unpredicted noncanonical base pairing and stacking interactions. The result is a wide major groove that perfectly complements the surface of the protein in size and shape. The most striking feature of the complex is that RNA mimics the DNA B form such that the chemistry of the core RNA/p50 RHR complex interface is essentially identical to that of the κB-DNA/p50 Rel homology domain interface. Thus, although the RNA aptamer bears no obvious sequence homology to κB-DNA (Fig. 10), it binds p50 with striking similarity to κB-DNA. It remains unknown whether there are natural RNA partners for NF-κB TFs. What is clear from the study of selected anti-NF-κB RNA aptamers is that there is no obstacle to identifying RNA ligands whose affinity for DNA-binding proteins rivals or exceeds that of the natural DNA partner.

### Anti-TATA-binding protein

RNA aptamers against TATA-binding protein (TBP) have been described in two reports [131,132]. TBP is a critical basal TF that associates with the core promoter and acts with other factors to initiate gene transcription. TBP recruitment is the first and generally the rate-limiting step of transcription initiation by all three types of eukaryotic RNA polymerases [133,134]. TBP binds to the minor groove of the TATA box sequence [135,136].

In the first study [132], RNA aptamers against yeast TBP were identified by *in vitro* selection. After 11 selection cycles, no dominant consensus sequence was observed. Nonetheless, subsequent validation experiments suggested that the recovered RNAs specifically bound to TBP. Three aptamers were chosen for further affinity characterization by electrophoretic gel mobility shift assay. Equilibrium dissociation constants ranged between ∼3 and 10 nM. The aptamers were shown to compete with TBP for a TATA-binding sequence in double-stranded DNA, suggesting that the aptamers target the DNA-binding surface of TBP. The RNA aptamers were then incubated with multiple TBP•TATA-containing complexes to explore their disruptive activity. These data provided insight into the dynamics of TBP interactions during transcription reinitiation on a relevant kinetic time scale, and it was proposed that RNA aptamers could be used as tools to dissect transcription initiation mechanisms.

**FIG. 11.**
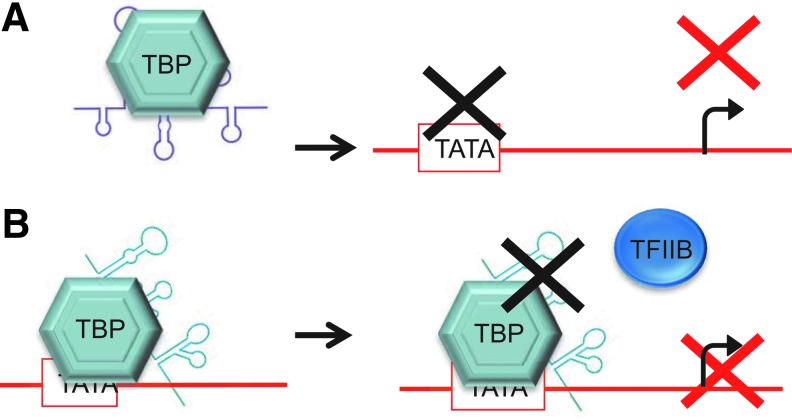
Proposed inhibition mechanism for two anti-TATA-binding protein (TBP) RNA aptamers. **(A)** Aptamer selected against TBP DNA-binding site acts as a competitive inhibitor preventing transcription by sequestering TBP. **(B)** Aptamers selected against a TBP-TATA complex are proposed to bind the TBP surface, preventing its interaction with other factors, for example, TFIIB, hence suppressing transcription.

The second study [131] involved *in vitro* selections against *Drosophila* TBP in free and TATA-bound forms. Using RNA libraries from 4 cycles of selection against TBP (prior study), 12 further parallel selection rounds were performed against TBP or a TATA•TBP complex. Interestingly, no sequence was found in common between either the selected pools or the pool created in the first study. Aptamers from both selections were studied in transcription initiation assays *in vitro*. It was proposed that aptamers from selections against free TBP target the DNA-binding surface of TBP and inhibit TBP binding to TATA box. In contrast, aptamers from the TATA•TBP selection were proposed to bind other surfaces and disrupt TBP interactions with other factors, thus preventing formation of the transcription initiation complex (Fig. 11). No further characterization of anti-TBP aptamers has been reported. If issues of delivery or endogenous production of such agents could be resolved, it would be interesting to compare effects of aptamer inhibition of TBP with those obtained with small molecules such as tallimustine, which bind TA-rich DNA and inhibits its interaction with TBP [137].

### Anti-heat shock factor

RNA aptamers have been selected against heat shock factor (HSF) with the goal of dissecting transcription activation mechanisms *in vivo* and *in vitro* [138]. HSF is a highly conserved TF crucial for the stress response [139] and aging [140] of eukaryotic cells. HSF regulates genes involved in energy generation, signal transduction, vesicular transport, and chaperone function [141]. HSF functions as a homotrimer and has a highly conserved DNA-binding domain.

**FIG. 12.**
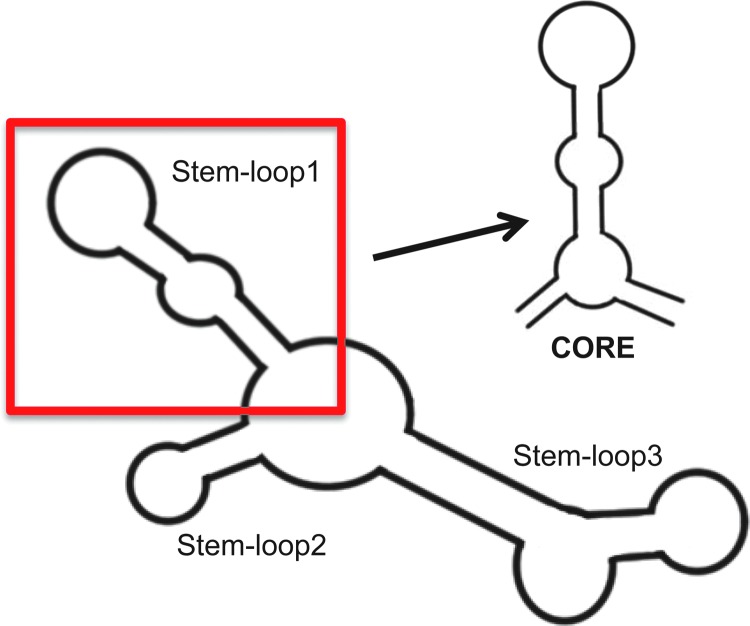
Schematic of anti-HSF1 secondary structure (in *red*) minimal structure required for binding and inhibition.

A dominant anti-HSF RNA aptamer was 90 nucleotides in length and secondary structure prediction suggested a three-way junction radiating three different stem-loops (Fig. 12). It was found that the minimal structure required for binding and inhibition was a 45-nucleotide RNA core. Full-length and core aptamers were characterized by equilibrium dissociation constants of 20–40 and 40–80 nM, respectively. Aptamer specificity was assessed by testing binding to other TF such as TBP, GAGA, and Gal4. Binding was not observed even at high protein concentrations. When exposed to lysates of SF9 cells that did and did not express HSF, aptamer complexes were only detected when HSF was present [138].

Clever *in vivo* studies were then designed using an aptamer expression system to rapidly generate high concentrations of a divalent version of anti-HSF, with a higher affinity for HSF (*K*_d_∼8 nM). Endogenous expression during *Drosophila* development produced phenotypes that closely resembled abnormalities that occur when HSP activity is reduced, particularly a notched wing phenotype (observed in ∼90% of flies expressing the divalent anti-HSF1 aptamer construct). It was further shown that abnormal phenotypes caused by aptamer inhibition of HSF1 could be suppressed upon HSF1 overexpression. Thus, these experiments convincingly demonstrated that anti-HSF1 aptamers can prevent HSF1 from activating gene expression under normal and stress conditions [142]. Since it has been noted that downregulation of HSF activity sensitizes cancer cells to anticancer drugs [143], anti-HSF1 aptamers could have a potential value in cancer therapy.

### Anti-runt-related transcription factor 1

RNA aptamers against the runt-related transcription factor 1 (RUNX1) TF have been reported. RUNX1 also known as acute myeloid leukemia 1 protein (AML1) is the α subunit of the core-binding factor (CBF) [144]. RUNX1 is one of the most important regulators of hematopoiesis, regulating transcription of a range of blood cell-specific genes [145,146]. RUNX1-deficient mice do not generate definitive hematopoietic cells and embryos die at developmental day 12 [147]. RUNX1 interacts with DNA through a 128 amino acid runt homology domain (RHD) localized at its N-terminus.

**FIG. 13.**
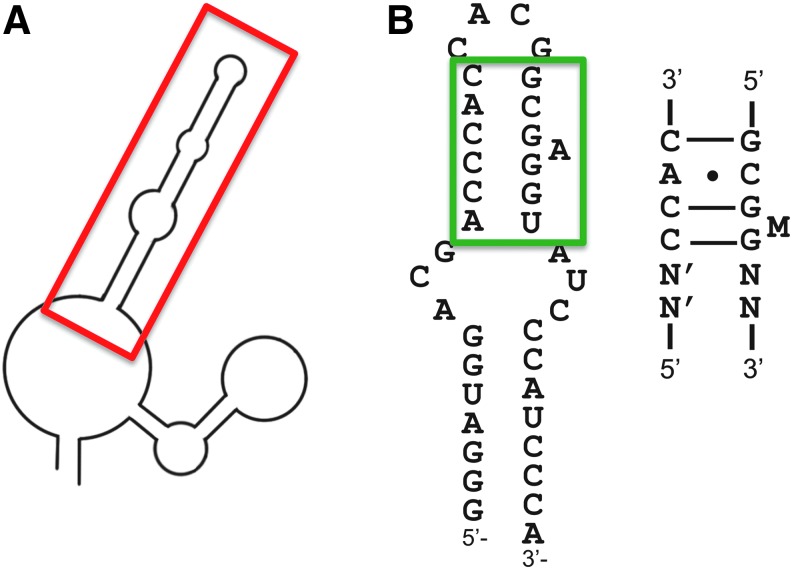
Anti-runt-related transcription factor 1 (RUNX1) aptamers. **(A)** Schematic of the secondary structure of a representative aptamer (minimal region for RUNX1 inhibition *boxed* in *red*). Adapted from Barton *et al.* [148]. **(B)** These proposed secondary structure of the minimal region hairpin (38 nucleotides, *boxed* in *green*) thought to mimic the Runt-binding double-stranded DNA element (RDE). Adapted from Fukunaga *et al.* [149].

A 2009 report describes 2′-fluoro-pyrimidine-modified RNA aptamers selected against a recombinant RHD-CBFβ complex using a random library containing 50 random nucleotides [148]. After 10 cycles of selection, the authors found aptamers with affinity for their target (*K*_d_ ∼ 100 nM). Surprisingly, when the authors tested aptamers with or without 2′-fluoro-modified pyrimidines, similar affinities were observed, suggesting that these modified nucleotides did not contribute to aptamer structure or sequence recognition by the protein. Competition experiments confirmed that anti-RHD-CBFβ RNA aptamers interfere with the formation of DNA-RHD-CBFβ complexes. Secondary structure prediction for these aptamers suggested that they contain a 5′ stem-loop that is strongly protected by RHD-CBFβ in footprint assays [148]. This stem-loop structure proved to be sufficient to inhibit RHD-CBFβ binding to RNA (Fig. 13A). Further experiments demonstrated that the aptamer was specific for RHD by using antibodies against the N- and C-terminal regions of RUNX1 and demonstrating a super shift of the aptamer complex in electrophoretic gel mobility shift assays.

RNA aptamers against Runt domain of RUNX1 were also described in a 2013 publication describing two distinct RNA libraries with 30 or 40 random positions [149]. After nine rounds of selection, all aptamers contained the conserved sequence motifs 5′CCAC3′ and 5′GCGMG3′ spaced by four to six nucleotides. The predicted secondary structures of these aptamers show a hairpin structure with internal loops. Enzymatic probing using single-strand-specific RNases corroborated this structure (Fig. 13B). A minimal structure of the main hairpin structure bound Runt with affinity similar to the full-length aptamer (equilibrium dissociation constant values of 1–3 nM). The NMR solution structure of a minimal aptamer of 22 nucleotides showed that the hairpin loop is contorted such that the motif contains an AH^+^-C mismatch and a base triple to adopt an unusual backbone structure that mimics the double-stranded DNA structure of the Runt recognition sequence [150]. A comparative study of RUNX1 bound to the aptamer motif and the runt domain corroborated these findings. The anti-RUNX1 RNA aptamer thus provides another remarkable example of the growing list of RNAs that mimic DNA architecture when binding to DNA-binding proteins.

## Conclusion

TFs are often deranged in disease, making them attractive targets for therapy. TFs occur in lower concentrations than other targets and form focal points in deregulated pathways [6]. Inhibition of deregulated TFs might eventually be achieved utilizing RNA aptamer inhibitors.

In this study we have reviewed several examples of RNAs that modulate the activity of TFs in various contexts. Our survey began with natural RNAs that modulate TF activity either resulting in inhibition of transcription (5S RNA, 6S RNA, 3′ UTR of cad, TSU, 7SK, anti-p53 RNA, and Gas5) or in modulation of transcription by binding to TFs or polymerases (NRSE-RNA, HDV, and SRA). Although unrecognized initially, the increasing inventory of natural RNA partners for DNA-binding proteins, including TFs and polymerases, suggests that there may be another level of gene expression regulation yet to be characterized. It is interesting to note that several of the known TFs modulated by RNA are zinc finger proteins (TFIIIA, REST, STAT1, TRA1, WT1) [98]. The DNA-binding domains of these proteins are structurally conserved [151–155], and seem to have evolved variants selective for RNA or DNA recognition, with opportunity for promiscuity. Thus, we believe that it is likely that many coding and noncoding RNAs may play roles as TF decoys or adapters [156].

We next reviewed examples of several artificially selected RNA aptamers against TFs. Such aptamers have typically been selected with the goal of creating tools for understanding protein–protein and protein–DNA interactions, and with an eye toward potential therapeutic use (anti-NF-κB, anti-TBP, anti-HSF1, anti-RUNX). The reported aptamers typically have high target affinity, often higher than their affinity for a cognate DNA-binding sequence. The aptamers are also typically characterized by high specificity and distinguish between isoforms and other related proteins. Most anti-TF aptamers engage in mimicry of double-stranded DNA. These characteristics together with low immunogenicity, small size, ease of synthesis and purification, and facile modification have made RNA aptamers attractive leads for therapeutic concepts [157,158].

Although RNA aptamers present obvious challenges in drug delivery, they can be encoded by synthetic genes whose expression can be controlled. Maximizing aptamer expression from transgenes has been overcome in two ways. Expression can be increased by creating expression systems in which aptamer multimer transcripts self-cleave using punctuating ribozyme sequences [142,159], or by inserting self-splicing introns with aptamer sequences into every copy of the ∼150 highly expressed rRNA genes using a specific homing endonuclease. This approach may help deliver high concentrations of desired aptamer with minimal collateral disruption [160], but requires transgenic technology.

Therapeutic aptamer drugs have been slow to develop. While a number of aptamers have completed various stages of preclinical development, only one aptamer (targeting vascular endothelial growth factor) completed phase III clinical trials and is now marketed for the treatment of age-related macular degeneration [10,161]. The existence of natural RNA aptamers for TF and other DNA-binding proteins points the way to a potential future for unnatural RNA aptamers for research and therapy.
